# Research on Ship-Radiated Noise Denoising Using Secondary Variational Mode Decomposition and Correlation Coefficient

**DOI:** 10.3390/s18010048

**Published:** 2017-12-26

**Authors:** Yuxing Li, Yaan Li, Xiao Chen, Jing Yu

**Affiliations:** School of Marine Science and Technology, Northwestern Polytechnical University, Xi’an 710000, China; chenxiao@mail.nwpu.edu.cn (X.C.); yujing@nwpu.edu.cn (J.Y.)

**Keywords:** variational mode decomposition (VMD), secondary variational mode decomposition (2VMD), correlation coefficient (CC), ship-radiated noise (SN), denoising

## Abstract

As the sound signal of ships obtained by sensors contains other many significant characteristics of ships and called ship-radiated noise (SN), research into a denoising algorithm and its application has obtained great significance. Using the advantage of variational mode decomposition (VMD) combined with the correlation coefficient for denoising, a hybrid secondary denoising algorithm is proposed using secondary VMD combined with a correlation coefficient (CC). First, different kinds of simulation signals are decomposed into several bandwidth-limited intrinsic mode functions (IMFs) using VMD, where the decomposition number by VMD is equal to the number by empirical mode decomposition (EMD); then, the CCs between the IMFs and the simulation signal are calculated respectively. The noise IMFs are identified by the CC threshold and the rest of the IMFs are reconstructed in order to realize the first denoising process. Finally, secondary denoising of the simulation signal can be accomplished by repeating the above steps of decomposition, screening and reconstruction. The final denoising result is determined according to the CC threshold. The denoising effect is compared under the different signal-to-noise ratio and the time of decomposition by VMD. Experimental results show the validity of the proposed denoising algorithm using secondary VMD (2VMD) combined with CC compared to EMD denoising, ensemble EMD (EEMD) denoising, VMD denoising and cubic VMD (3VMD) denoising, as well as two denoising algorithms presented recently. The proposed denoising algorithm is applied to feature extraction and classification for SN signals, which can effectively improve the recognition rate of different kinds of ships.

## 1. Introduction

In the practical measuring process, measured signals are often mixed with noise and useless signal components which come from the surrounding complex environment and the measurement equipment itself. The time domain waveforms of the polluted signals are often different from those of original signals, and it is not easy to identify the original signal from the polluted signal. In the frequency domain, the bandwidth of the clear signal and the noisy signal also partially or even completely overlap. In this case, traditional spectrum analysis techniques and linear filtering algorithms cannot effectively eliminate noise. Therefore, the question of how to eliminate noise from the polluted signal must be solved in the signal processing field [1,2].

There is a kind of common denoising algorithm, the basic ideas of which is to extract components of signal obtained by one signal decomposition algorithm, identify and remove noise components by screening criteria, then reconstruct the useful components. For this kind of denoising algorithm, the focus is on selecting signal decomposition algorithm and noise-screening criteria. For instance, the wavelet denoising algorithm [3,4] has been widely used in various fields, and a good effect is gained. However, using different types of wavelet functions and different numbers of decomposition have a great influence on denoising [5].

A kind of self-adaptive signal processing algorithm is empirical mode decomposition (EMD) [6,7], originally proposed by Huang et al. This is an absolutely data-driven and adaptive algorithm that depends on local characteristics of data in the time domain. EMD can decompose complex signal into intrinsic mode functions (IMFs), with each IMF indicating one oscillation mode of the complex signal. However, EMD faces problems of mode-mixing and end effects and also lacks mathematical demonstration. Ensemble EMD (EEMD) [8], as an algorithm that improves on EMD, can reduce the phenomena of modes overlap to some extent. A growing number of researchers are focusing on developing EMD and improved EMD algorithms, and these algorithms are widely employed in various fields, especially in mechanical fault diagnosis [9,10,11], medical science [12], meteorology [13], oceanography [14,15,16,17,18] and so on. Many denoising algorithms using EMD and improved EMD algorithms have been proposed. For example, high-frequency IMFs are regarded as noise IMFs; the rest of the IMFs are reconstructed for denoising [19]. Nevertheless, this denoising algorithm cannot completely eliminate noise components, and the reconstructed signal lacks some detailed information. Many denoising algorithms have been proposed to solve the problems of this denoising algorithm by the threshold for IMFs [20,21].

As a kind of non-recursive and self-adaptive signal-processing algorithm, variational mode decomposition (VMD) [22,23,24], originally put forward by Dragomiretskiy et al., can effectively decompose a multi-component signal into several bandwidth-limited IMFs. Every IMF has a corresponding central frequency updated in real-time. Compared with EMD and the improved EMD algorithms, VMD has not only a solid theoretical foundation, but also good robustness to noise. In the field of fault diagnosis, a new diagnosis algorithm based on VMD denoising is proposed in [25], which uses the IMFs obtained by VMD to reconstruct the IMFs according to the correlation coefficients (CCs) between IMFs and the original signal in order to realize denoising, and then extracts the bearing fault characteristics by means of a morphological difference filter to demodulate the signals after denoising, with simulated signal and experimental results showing the validity of the algorithm. In research [26], a new denoising algorithm based on the non-convex framework has been proposed. By comparing with wavelet denoising and 1-order total variation denoising algorithms, the validity is verified by analyzing the simulation signals and the vibration signals. In research [27], an adaptive denoising algorithm for a chaotic signal has been proposed by using independent component analysis (ICA) and EMD. In research [28], an adaptive denoising algorithm using a probability density function and VMD has been proposed, and a small mean-error square and a high signal-to-noise ratio prove the effectiveness of the denoising algorithm. These denoising algorithms also demonstrate the feasibility of EMD and VMD in signal denoising.

In this article, we proposed a new denoising algorithm for ship-radiated noise (SN) signals. We used VMD and CC to decompose the original signals into IMFs and identify noise IMFs, respectively; the decomposition number by VMD is equal to the number by EMD. According to the threshold of the CC, noise IMFs and useful IMFs can be distinguished effectively. Then, the first denoising can be realized by reconstructing useful IMFs. Secondary denoising can be accomplished by repeating the above steps of decomposition, mode-identification and reconstruction. The final result of denoising is determined according to the CC between IMFs and the original signal. Simulation results indicate that the proposed denoising algorithm based on the secondary VMD (2VMD) and CC is better than existing denoising ones. The proposed 2VMD denoising algorithm is used to feature extraction and classification for SN signals, which can effectively improve the recognition rate of different kinds of ships.

The outline of the article is as follows. Section 2 provides the background to VMD, CC and the evaluation criterion; a review of the proposed 2VMD denoising algorithm is presented in Section 3; in Section 4 and Section 5, the proposed 2VMD denoising algorithm is used to simulation data and SN signals respectively; finally, the last section is the conclusions.

## 2. Background

### 2.1. Variational Mode Decomposition (VMD)

In the VMD algorithm, IMFs are defined as amplitude-modulated–frequency-modulated (AM–FM) signals, which are given by:

(1) $$u_{k}(t)=A_{k}(t)\cos(\phi_{k}(t)),$$

where t and $A_{k}(t)$ represent time and the envelope of IMF; and $\phi_{k}(t)$ and $u_{k}(t)$ denote the phase and the IMFs. IMFs have center frequencies and limited bandwidths. The decomposition process is the constrained variational problem, which is given by:

(2) $$\underset{\left\{u_{k}\},\{w_{k}\right\}}{\min}\left\{\sum_{k=1}^{K}{\left\|\partial t[(\delta (t)+\frac{j}{\pi t})*u_{k}(t)]e^{-jwkt}\right\|}_{2}^{2}\right\}\;subject\;to\sum_{k=1}^{K}u_{k}=s,$$

where s is the original signal; K represents the number of IMFs; and $u_{k}$ and $w_{k}$ are the IMF and the center frequency for each IMF. The constrained variational problems in Equation (2) can be addressed by the penalty factor α and the lagrangian multiplier λ. The augmented lagrangian is expressed as:

(3) $$\begin{array}{cc} L(\{u_{k}\},\{w_{k}\},\lambda ) & =\alpha \sum_{k=1}^{K}{\left\|\partial t[(\delta (t)+\frac{j}{\pi t})*u_{k}(t)]e^{-jwkt}\right\|}_{2}^{2}\\ & +{\left\|f(t)-\sum_{k=1}^{K}u_{k}(t)\right\|}_{2}^{2}+\langle \lambda (t),f(t)-\sum_{k=1}^{K}u_{k}(t)\rangle . \end{array}$$

The alternating direction multiplier method (ADMM) is used to obtain the saddle points, then the $u_{k}$, $w_{k}$ and λ are updated in the frequency domain, which is given by:

(4) $${\hat{u}}_{k}^{n+1}(w)=\frac{\hat{f}(w)-\sum_{i<k}\hat{u}i^{n}(w)-\sum_{i>k}\hat{u}i^{n}(w)+\frac{{\hat{\lambda }}^{n}(w)}{2}}{1+2\alpha {\left(w-w_{k}^{n}\right)}^{2}},$$

(5) $$w_{k}^{n+1}=\frac{\int_{0}^{\infty }w{\left|{\hat{u}}_{k}^{n+1}\right|}^{2}dw}{\int_{0}^{\infty }{\left|{\hat{u}}_{k}^{n+1}\right|}^{2}dw},$$

(6) $${\hat{\lambda }}^{n+1}(w)={\hat{\lambda }}^{n}(w)+\tau \left(\hat{f}(w)-\sum_{k}{\hat{u}}_{n}^{n+1}(w)\right).$$

The stop condition is as follows:

(7) $${\sum_{k}\left\|{\hat{u}}_{k}^{n+1}-{\hat{u}}_{k}^{n}\right\|}_{2}^{2}/{\left\|{\hat{u}}_{k}^{n}\right\|}_{2}^{2}<e,$$

where e represents convergence accuracy. The specific process of VMD is summarized as follows:

- Initialize $\left\{{\hat{u}}_{k}^{1}\right\}$, $\left\{w_{k}^{1}\right\}$, ${\hat{\lambda }}^{1}$ and *n* = 0.
- Update the value of $\left\{{\hat{u}}_{k}^{n+1}\right\}$, $\left\{w_{k}^{n+1}\right\}$ and ${\hat{\lambda }}^{n+1}$ according to Equations (4)–(6).
- Judge whether or not $u_{k}$ meets the convergence condition (7).

Repeat the steps of updating parameters until the stopped condition is satisfied.

The uniformly spaced distribution for initialization of center frequency is expressed as:

(8) $$w_{k}^{0}=\frac{k-1}{2k}\begin{array}{cc} , & \end{array}k=1,\cdots ,K,$$

and the zero initial can be expressed as:

(9) $$w_{k}^{0}=0\begin{array}{cc} , & \end{array}k=1,\cdots ,K.$$

In addition, K is equal to the decomposition level by EMD. The zero initial is used in this paper.

### 2.2. Correlation Coefficient (CC)

The correlation coefficient (CC), as a parameter of statistical relationships, can measure the degree of dependence and correlation. In this paper, the formula of CC is shown as the following:

(10) $$r=\frac{E(ui.f)-E(ui)E(f)}{\sqrt{D(ui)}\sqrt{D(f)}},$$

where f and $u_{i}$ represent the original signal and IMF obtained by mode decomposition; D and E correspond to mathematical expectation and variance; and r represents the CC between the IMF and original signal. High values (close to 1) indicate a strong degree of dependence and correlation. Instead, the closer that this value is to −1, the more inverse the relationship value is. The relationship between CC and correlation is shown in Table 1. If CCs between the original signal and IMFs by VMD are within the range of moderate correlation or strong correlation, IMFs contain useful components. Therefore, the CC threshold is within the range of weak correlation.

**Figure 1:**
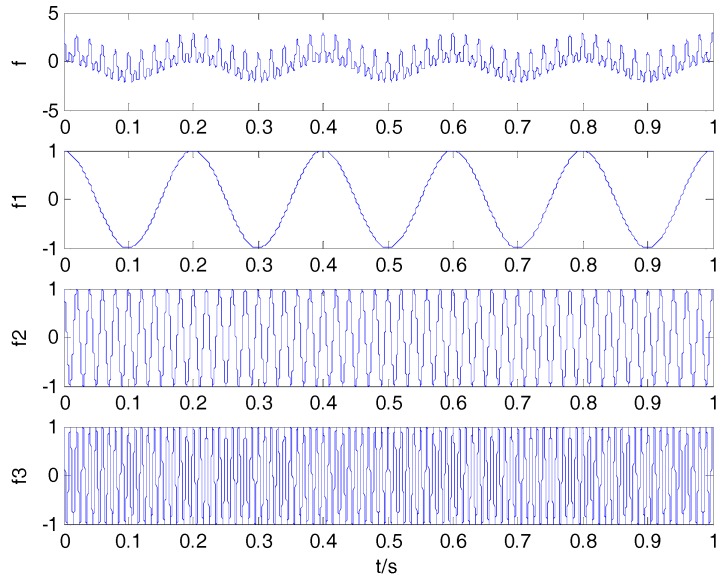
Simulation signals.

**Figure 2:**
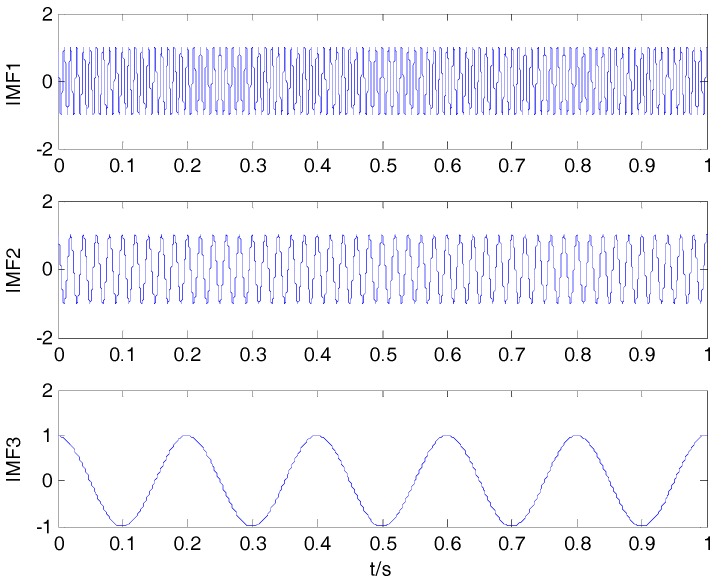
Decomposition result by variational mode decomposition (VMD).

A simulation example should make this easier to understand, with the simulation signals as follows:

(11) $$\left\{ \begin{array}{l} f_{1}(t)=\cos(10\pi t)\\ f_{2}(t)=\cos(100\pi t)\\ f_{3}(t)=\cos(200\pi t)\\ f(t)=f_{1}(t)+f_{2}(t)+f_{3}(t) \end{array}\right.,$$

where $f_{1}(t)$, $f_{2}(t)$ and $f_{3}(t)$ represent the three components of f(t). Three decomposition algorithms are used to decompose f(t). The original signals are presented in Figure 1. The decomposition result of VMD is shown in Figure 2.

As can be seen in Figure 1 and Figure 2, the decomposition result using VMD is similar to the component of the simulation signal. CCs between the simulation signal and corresponding IMFs are shown in Table 2. By comparison with EMD and EEMD algorithms, the CCs between simulation signal and corresponding IMFs by VMD are closer to the true values in Table 2. This shows that the VMD algorithm can better reflect the correlation.

### 2.3. Evaluation Criteria for Denoising Algorithm

The denoising effects of different decomposition algorithms are compared. Therefore, two evaluation criteria for denoising algorithms are given as follows:

(12) $$SNR=10\log10\left(\frac{\left\|f\right\|}{\left\|\hat{f}-f\right\|}\right),$$

(13) $$RMSE=\sqrt{\frac{\left\|\hat{f}-f\right\|}{N}},$$

where f is original signal; $\hat{f}$ is the denoising result; and N represents signal length. Signal-to-noise ratio (SNR) and root mean square error (RMSE) are the evaluation criteria for denoising, respectively.

## 3. Denoising Algorithm Using Secondary VMD (2VMD) and CC

**Figure 3:**
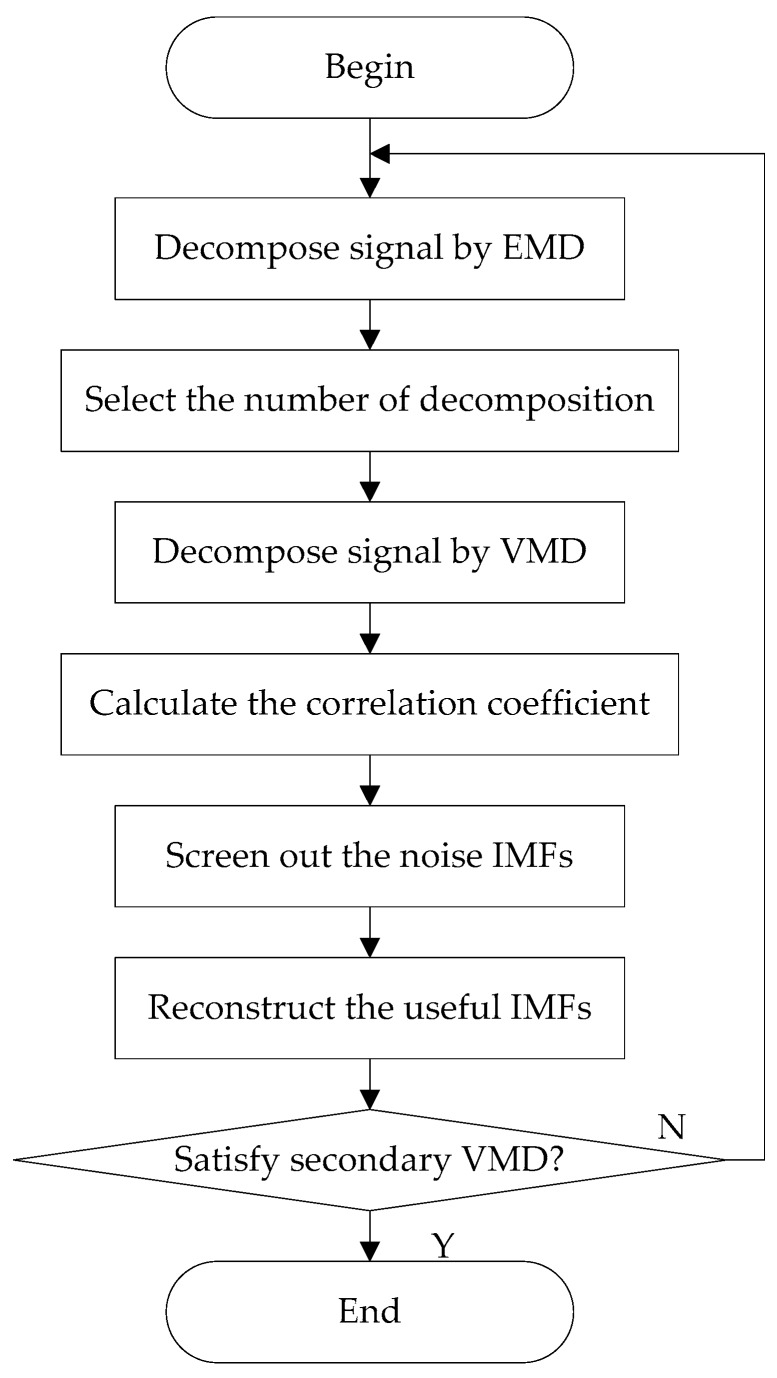
The flow chart of proposed secondary variational mode decomposition (2VMD) denoising algorithm.

A 2VMD denoising algorithm using VMD and CC is designed in Figure 3. The experimental procedures are as follows:

Step 1: The target signal is decomposed by EMD, and the decomposition number by VMD is equal to the number by EMD;
Step 2: Calculate the CCs between the original signal and IMFs by VMD, screen out the noise IMFs according to CC threshold. Through abundant simulation experiments, the CC threshold is fixed at 0.2 in this paper;
Step 3: Reconstruct the useful IMFs by removing noise IMFs. After the reconstruction, the first denoising is completed;
Step 4: Judge the times decomposition satisfies the 2VMD or not;
Step 5: If 2VMD is not satisfied, the first reconstructed result is regarded as the input signal, then repeat Steps 1–3 to complete the whole denoising process; if it is satisfied, the reconstructed signal is regarded as the final denoising result.

## 4. Test with Numerical Simulation Signal

The line spectrum is the important information of the SN signals, and it provides a basis for ship detection and tracking. The periodic signal can be used as a line spectrum model. Therefore, three simulation experiments have been carried out in Section 4.1, Section 4.2 and Section 4.3; the different input SNRs and the times of decomposition by VMD for the three simulation signals are also discussed in Section 4.4. To further prove the effectiveness of the proposed denoising algorithm, in Section 4.5 we compare it with two denoising algorithms presented recently using the same simulation signals.

### 4.1. Simulation 1

**Figure 4:**
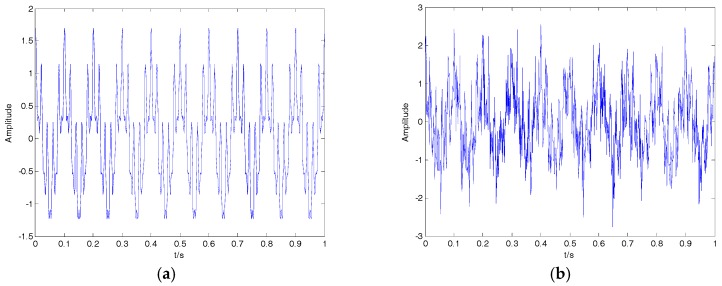
(**a**) The clear signal; (**b**) the noisy signal.

**Figure 5:**
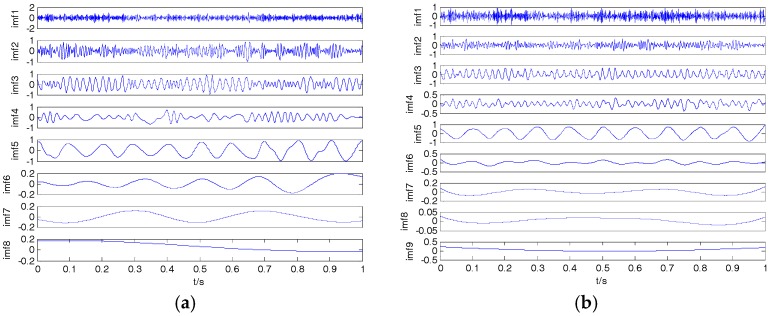

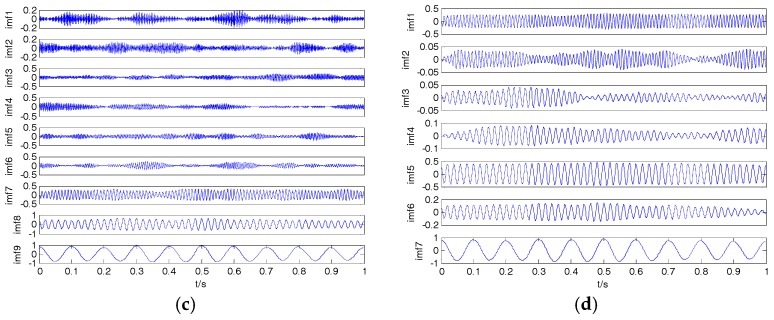
The decomposition result of empirical mode decomposition (EMD), ensemble EMD (EEMD), VMD and 2VMD for the noisy signal. (**a**) EMD; (**b**) EEMD; (**c**) VMD; (**d**) 2VMD.

The simulation signal s is composed of three different frequency and amplitude cosine signals, and 0.5 times standard Gaussian white noise n is added to get the noisy signal y. The simulation signals are as follows:

(14) $$\left\{ \begin{array}{l} s=0.8\cos(2\pi f_{1}t)+0.6\cos(2\pi f_{2}t)+0.3\cos(2\pi f_{3}t)\\ n=0.5randn(t)\\ y=s+n \end{array}\right.,$$

where $f_{1}=10$, $f_{2}=50$ and $f_{3}=100$ represent the three frequencies of clear signal s; and y is the noisy signal containing both s and n. The time-domain waveform for clear signal and noisy signal is shown in Figure 4. The decomposition result of the EMD, EEMD, VMD and 2VMD for a noisy signal are presented in Figure 5.

As seen in Figure 5, the number of IMFs by EMD is 9 (containing residue), and the number of VMD should be equal to the number of EMD, so we can set K=9 for VMD and set K=7 for 2VMD. The CCs between the noisy signal and IMFs by VMD and 2VMD are shown in Table 3.

**Figure 6:**
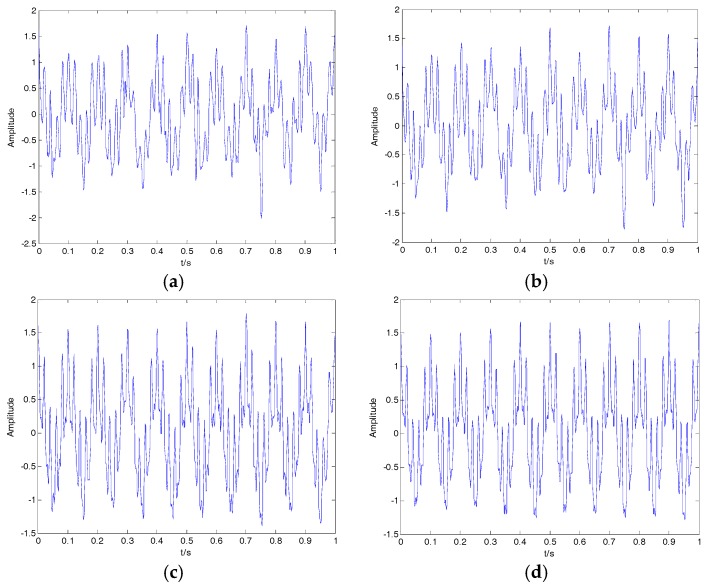
The denoising results of EMD, EEMD, VMD and 2VMD for the noisy signal. (**a**) EMD; (**b**) EEMD; (**c**) VMD; (**d**) 2VMD.

As seen in Table 3, the number of useful IMFs by VMD is three according to the CC threshold; the 2VMD can further remove noise IMFs (IMF2, IMF3 and IMF4) by the CC threshold. The denoising results of EMD, EEMD, VMD and 2VMD for the noisy signal are shown in Figure 6. The SNR and RMSE for EMD, EEMD, VMD and 2VMD denoising are shown in Table 4.

As seen in Figure 6, the denoising results of EMD and EEMD are obviously different from the clear signal; the denoising results of VMD and 2VMD are close to the clear signal. To compare the performance of different denoising algorithms, the SNR and RMSE are listed in Table 4. As can be seen in Table 4, EEMD denoising is superior to EMD denoising, and VMD denoising is better than EEMD denoising; 2VMD denoising is the best denoising algorithm which has high SNR and low RMSE.

### 4.2. Simulation 2

**Figure 7:**
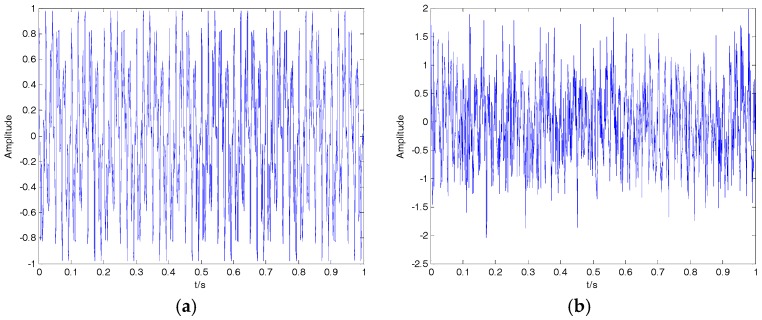
The time-domain waveform for the clear signal and noisy signal. (**a**) The clear signal; (**b**) the noisy signal.

**Figure 8:**
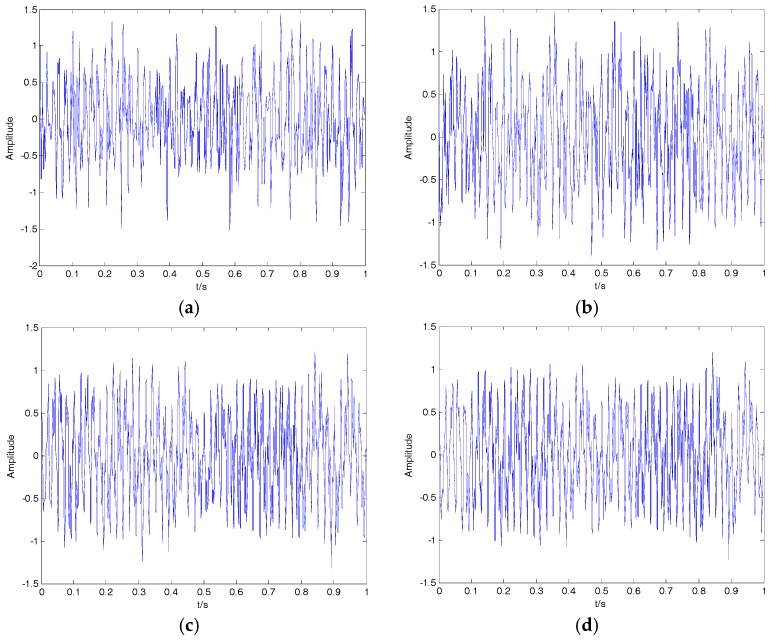
The denoising results of EMD, EEMD, VMD and 2VMD for the noisy signal. (**a**) EMD; (**b**) EEMD; (**c**) VMD; (**d**) 2VMD.

The simulation signal s is composed of frequency-modulated signal and sine signal, and 0.5 times standard Gaussian white noise n is added to get the noisy signal y. The simulation signals are as follows:

(15) $$\left\{ \begin{array}{l} s=0.6\cos(2\pi f_{1}t+0.8\sin(2\pi f_{2}t))+0.4\sin(2\pi f_{3}t)\\ n=0.5randn(t)\\ y=s+n \end{array}\right.,$$

where $f_{1}=50$, $f_{2}=40$ and $f_{3}=150$ represent the three frequencies of clear signal s; and y is the noisy signal containing both s and n. The time-domain waveform for the clear signal and noisy signal is shown in Figure 7, with the clear signal submerged in Gaussian white noise. The denoising results of EMD, EEMD, VMD and 2VMD are shown in Figure 8, and the SNR and RMSE for EMD, EEMD, VMD and 2VMD denoising are shown in Table 5. As seen in Figure 8 and Table 5, the 2VMD denoising algorithm with high SNR and low RMSE is also the most effective denoising algorithm.

### 4.3. Simulation 3

**Figure 9:**
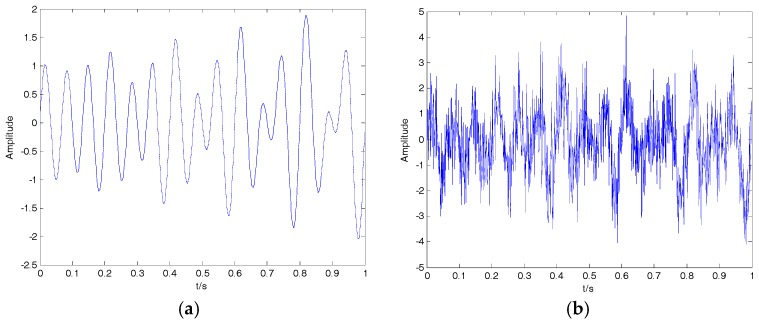
(**a**) The clear signal; (**b**) the noisy signal.

**Figure 10:**
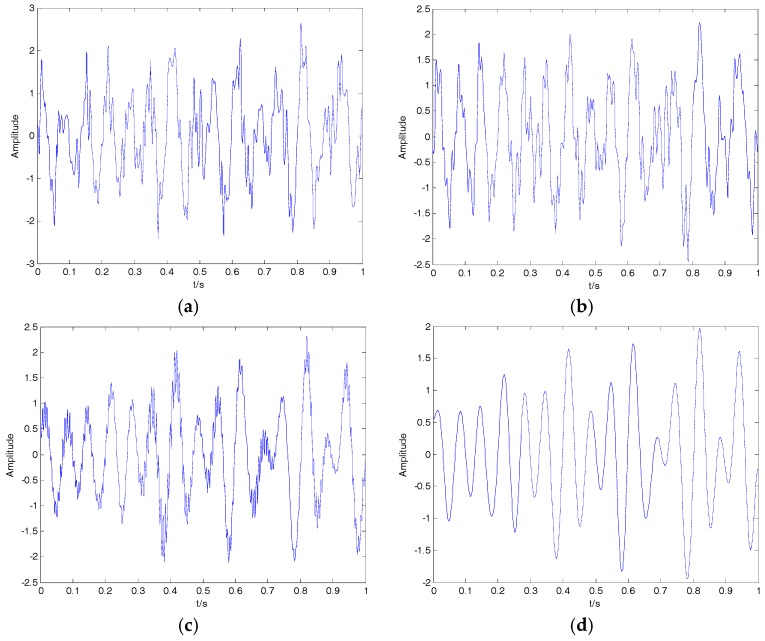
The denoising results of EMD, EEMD, VMD and 2VMD for the noisy signal. (**a**) EMD; (**b**) EEMD; (**c**) VMD; (**d**) 2VMD.

The simulation signal s is composed of an amplitude-modulated signal and sine signal, and standard Gaussian white noise n is added to get the noisy signal y. The simulation signals are as follows:

(16) $$\left\{ \begin{array}{l} s=2\sin(2\pi f_{1}t)\sin(2\pi t/f_{2})+\sin(2\pi f_{3})\\ n=randn(t)\\ y=s+n \end{array}\right.,$$

where $f_{1}=10$, $f_{2}=10$ and $f_{3}=15$ represent the three frequencies of clear signal s; and y is the noisy signal containing both s and n. The time-domain waveform for the clear signal and noisy signal is shown in Figure 9; the clear signal cannot be distinguished from the noisy signal. The denoising results of EMD, EEMD, VMD and 2VMD for the noisy signal are shown in Figure 10, and the SNR and RMSE for EMD, EEMD, VMD and 2VMD denoising are shown in Table 6. As seen in Figure 10 and Table 6, the 2VMD denoising algorithm with high SNR and low RMSE, which is smooth and close to the clear signal, is the most effective denoising algorithm.

### 4.4. Different Input Signal-to-Noise Ratio (SNR) and Times of Decomposition by VMD

**Figure 11:**
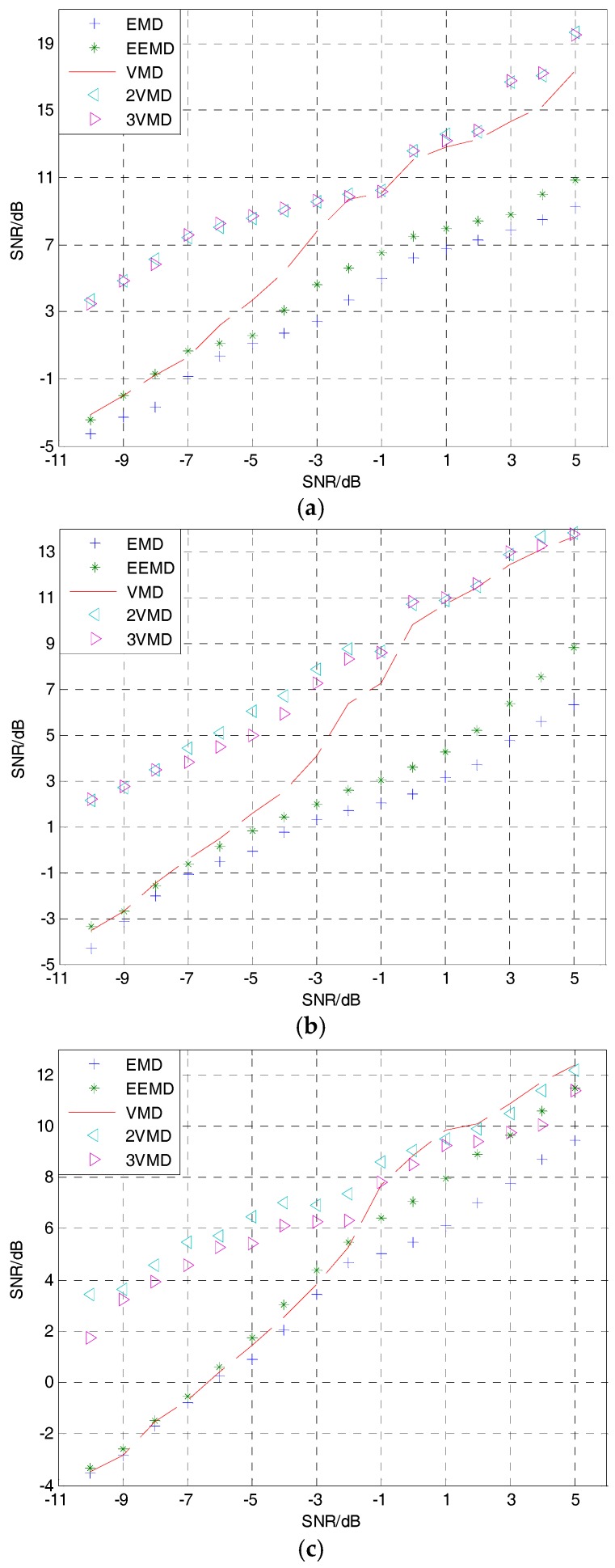
The denoising results of EMD, EEMD, VMD, 2VMD and cubic VMD (3VMD) for different simulation signals. (**a**) The denoising results of simulation 1; (**b**) the denoising results of simulation 2; (**c**) the denoising results of simulation 3.

To further prove the suitability of the proposed 2VMD algorithm, the denoising effect is compared under different input SNRs and times of decomposition by VMD for the simulation signals in Section 4.1, Section 4.2 and Section 4.3. Input SNRs range from −10 dB to 5 dB, and the times of decomposition for VMD range from 1 to 3. Figure 11 shows the plots of input SNRs versus output ones for different denoising algorithms and simulation signals, where each output SNR is calculated by using the mean of 100 times. As can be seen in Figure 11, the output SNRs of the 2VMD and cubic VMD (3VMD) denoising algorithms in most cases are higher than the others, especially in the case of low-input SNRs, which are more suitable for SN signal denoising. However, 2VMD denoising has the advantage of low computational cost over 3VMD denoising.

### 4.5. Comparison with Denoising Algorithms Presented Recently

**Figure 12:**
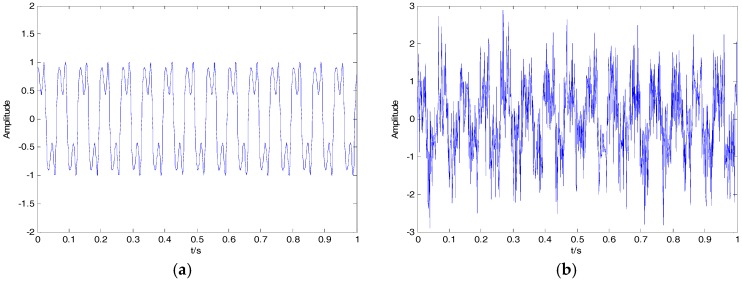
(**a**) The clear signal; (**b**) the noisy signal.

In recent research [26,29], two denoising algorithms have been proposed for the vibration signal and SN signal, respectively. The same simulation signals in [26] are as follows:

(17) $$\left\{ \begin{array}{l} x=x_{1}+x_{2}\\ x_{1}=\sin(30\pi t+\cos(60\pi t)) \end{array}\right.$$

where $x_{1}$ is a typical modulating signal; $x_{2}$ is Gaussian white noise, whose mean value and variance are 0 and 0.5, respectively. The clear signal $x_{1}$ and noisy signal $x_{2}$ are shown in Figure 12. The SNRs for different variances of Gaussian white noise and different denoising algorithms are shown in Table 7. As can be seen in Table 7, the proposed algorithm has high SNR for different variances of Gaussian white noise compared with the wavelet denoising and the denoising algorithms presented recently in [26,29].

## 5. Application in Feature Extraction for Ship-Radiated Noise (SN)

First, three kinds of SN signals are performed by the proposed 2VMD denoising algorithm; then, the features of SN signals are extracted by the feature extraction algorithm in [16]; finally, the classification results before and after denoising are compared.

### 5.1. Denoising of SN

**Figure 13:**
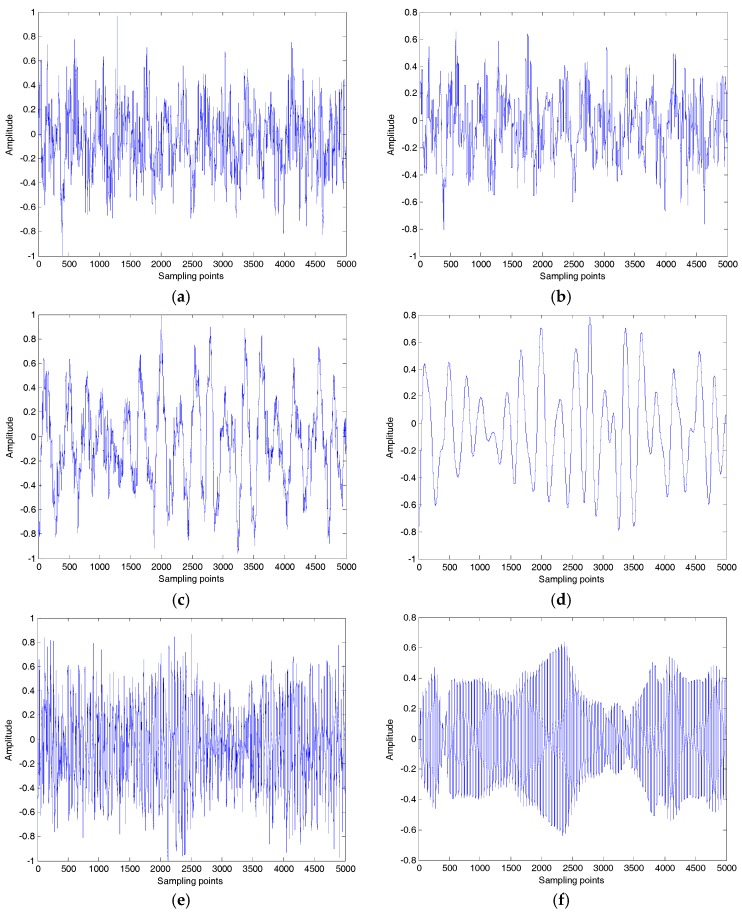
Three kinds of ship-radiated noise (SN). (**a**) Ship 1 without denoising; (**b**) Ship 1 after denoising; (**c**) Ship 2 without denoising; (**d**) Ship 2 after denoising; (**e**) Ship 3 without denoising; (**f**) Ship 3 after denoising.

Three kinds of SN signals, which are the same as the signals in [16], were recorded using calibrated omnidirectional hydrophones at a depth of 29 m in the South China Sea. During recording, there were no observed disturbances from biological or man-made sources. The distance between the ship and hydrophone was about 1 km. The sampling frequency and sampling points were set as 44.1 kHz and 5000, respectively. The samples were normalized to get the time-domain waveform for three kinds of SN signals shown in Figure 13a,c,e. The denoising results for three kinds of SN signals by the proposed denoising algorithm are shown in Figure 13b,d,f.

### 5.2. Feature Extraction of SN

**Figure 14:**
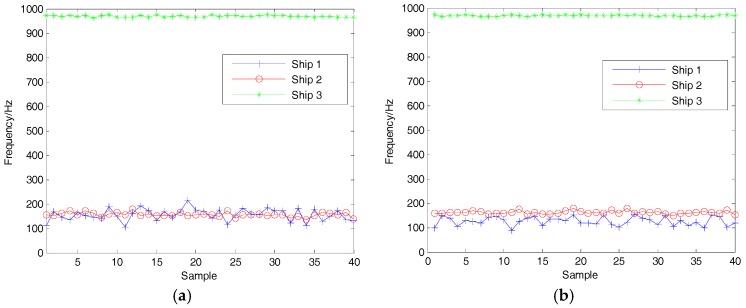
The center frequency of the IMF with the highest energy (EIMF). (**a**) without denoising; (**b**) after denoising.

According to the research in [16], three kinds of SN signals after denoising are decomposed by VMD. It is then easy to obtain the IMF with the highest energy (EIMF) by calculation, and the center frequency of EIMF is regarded as characteristic parameter in this paper. Forty samples for each kind of SN were selected to calculate the center frequency of the EIMF. The center frequency distribution of EIMF before and after denoising is shown in Figure 14. The proposed denoising algorithm is useful for distinguishing the first and second kinds of ships.

### 5.3. Classification

To further prove the effectiveness of the proposed 2VMD denoising algorithm, the center frequencies of EIMF are classified by a support vector machine (SVM), and the polynomial kernel function is used for training and identifying. The classification results of train and test samples are shown in Table 8 and Table 9. As shown in Table 8 and Table 9, the accuracy of ship 3 is 100%. However, the recognition rates of Ship 1 and Ship 2 have been improved significantly. The accuracy after denoising is 95.67%, which is obviously superior to that before denoising.

## 6. Conclusions

In order to achieve denoising of SN signals, a hybrid secondary denoising algorithm is proposed in this article. The proposed denoising algorithm employs 2VMD and CC. The target signal is decomposed using VMD, and the CC threshold is used to determine the useful IMFs. Through abundant simulation experiments and analytical comparisons, the proposed denoising algorithm demonstrated its superiority and the following contributions:

(1) A secondary VMD algorithm for denoising is put forward for the first time in this paper.
(2) A novel denoising algorithm is proposed using 2VMD and CC for the SN signal in the field of underwater acoustic signal processing.
(3) Compared with EMD and EEMD, the CCs between the simulation signal and its IMFs using VMD are closer to the true values. This shows that the VMD algorithm can better reflect the correlation.
(4) Compared with EMD, EEMD and VMD denoising, the proposed denoising algorithm is a better denoising algorithm which has a high SNR and low RMSE by numerical simulations.
(5) Compared with the different input SNRs and the times of decomposition by VMD, the proposed 2VMD denoising algorithm has high SNRs for different simulation signals, especially in the case of low-input SNRs. In addition, the proposed 2VMD denoising algorithm is superior to the two denoising algorithms presented recently in [26,29].
(6) Using the proposed 2VMD denoising algorithm and the feature extraction method in [16], the dominant frequency information is extracted. Compared with the feature extraction algorithm without denoising, the experimental results indicate that the proposed 2VMD denoising algorithm can effectively improve the recognition rate of different kinds of ships.

## Figures and Tables

**Table 1:** The relationship between correlation coefficient (CC) and correlation.

	No Correlation	Weak Correlation	Moderate Correlation	Strong Correlation
CC	0~0.1	0.1~0.3	0.3~0.5	0.5~1

**Table 2:** The CCs between simulation signal and corresponding intrinsic mode functions (IMFs).

Simulation Signals		EMD Algorithm		EEMD Algorithm		VMD Algorithm	
$f_{3}(t)$	0.5775	IMF1	0.6040	IMF5	0.5854	IMF1	0.5772
$f_{2}(t)$	0.5775	IMF2	0.5756	IMF6	0.6164	IMF2	0.5775
$f_{1}(t)$	0.5775	IMF3	0.5774	IMF8	0.5773	IMF3	0.5775

**Table 3:** The CCs between the noisy signal and IMFs by VMD and 2VMD.

	IMF 1	IMF 2	IMF 3	IMF 4	IMF 5	IMF 6	IMF 7	IMF 8	IMF 9
VMD	0.1438	0.1336	0.1592	0.1465	0.1583	0.1479	0.2417	0.4378	0.6721
2VMD	0.2205	0.0922	0.0927	0.0913	0.4204	0.3935	0.6673	−	−

**Table 4:** The signal-to-noise ratio (SNR) and root mean square error (RMSE) for EMD, EEMD, VMD and 2VMD denoising.

	Noisy Signal	EMD	EEMD	VMD	2VMD
SNR/db	2.959	8.133	9.33	15.01	17.34
RMSE	0.9797	0.8353	0.8246	0.1508	0.03072

**Table 5:** The SNR and RMSE for EMD, EEMD, VMD and 2VMD denoising.

	Noisy Signal	EMD	EEMD	VMD	2VMD
SNR/db	0.0827	2.5348	3.6687	10.3561	11.4758
RMSE	0.9335	0.7496	0.3384	0.2402	0.0626

**Table 6:** The SNR and RMSE for EMD, EEMD, VMD and 2VMD denoising.

	Noisy Signal	EMD	EEMD	VMD	2VMD
SNR/db	−1.467	4.898	6.335	8.306	9.412
RMSE	1.258	0.8652	0.8473	0.3065	0.1601

**Table 7:** The SNRs after denoising.

Algorithms	Variances		
	0.4	0.5	0.6
The proposed 2VMD denoising algorithm	13.12	12.34	11.87
The denoising algorithm in [27]	12.57	11.63	11.21
The denoising algorithm in [24]	12.13	11.31	10.28
The wavelet denoising algorithm	10.32	9.328	8.315
The noisy signal	0.9762	−0.05218	−0.9215

**Table 8:** The center frequency classification results without denoising.

Ship	Train Sample		Test Sample		Accuracy
	Sample	Accuracy	Sample	Accuracy	
1	100	64%	100	66%	68.5%
2	100	38%	100	43%	
3	100	100%	100	100%	

**Table 9:** The center frequency classification results after denoising.

Ship	Train Sample		Test Sample		Accuracy
	Sample	Accuracy	Sample	Accuracy	
1	100	94%	100	93%	95.67%
2	100	95%	100	92%	
3	100	100%	100	100%	
